# Health and environmental hazards of shape memory polymer used in orthodontic aligners – a scoping review

**DOI:** 10.2340/biid.v13.45225

**Published:** 2026-01-07

**Authors:** Sweety Agrawal, Mithun K. Naik, Dilshad Umar, Sandeep Shetty

**Affiliations:** Department of Orthodontics and Dentofacial Orthopaedics, Yenepoya Dental Orthodontics College, Yenepoya (Deemed to be University), Mangalore, Karnataka, India

**Keywords:** Clear aligners, shape memory aligners, SMP, 3D-printing aligner, orthodontic aligners, digital orthodontics, biocompatibility, cytotoxicity, environmental impact

## Abstract

**Background:**

Shape memory polymers (SMPs) are gaining traction in orthodontics, particularly in clear aligners, due to their stimulus-responsive behavior and potential to improve treatment outcomes. However, their use in biomedical devices raises questions about biocompatibility and environmental sustainability.

**Objectives:**

This scoping review aims to map current evidence on the health and environmental risks of SMPs used in orthodontic aligners, identify knowledge gaps, and guide future research.

**Search methods:**

A systematic search of PubMed, Scopus, Web of Science, Embase, ProQuest, and Cochrane databases was conducted, focusing on studies from the past decade. Search terms included SMPs, orthodontic aligners, toxicity, biodegradability, and environmental impact.

**Selection criteria:**

Eligible studies involved original in vitro, in vivo, clinical, or environmental research related to SMPs in orthodontic applications.

**Data collection and analysis:**

Key information from each study was extracted using a standardized Excel spreadsheet by one reviewer (SA) and validated by another (MKN). Extracted data included study design, polymer type, health and environmental risks, and conclusions. Due to heterogeneity, results were synthesized narratively.

**Results:**

Sixteen studies met the inclusion criteria. Evidence on SMP biocompatibility is emerging but limited. Certain SMP formulations released residual monomers and degradation products with potential cytotoxic or systemic effects. Environmental concerns included low degradability, accumulation of polymer waste, and lack of recycling strategies. Notably, there was a lack of long-term clinical data and environmental life-cycle analyses.

**Conclusions:**

While SMPs offer promise in orthodontics, their safety and environmental impacts are insufficiently studied. Future research should focus on standardized toxicological testing, long-term evaluations, and sustainable material development.

**Registration and Conflicts:**

This scoping review was prospectively registered in PROSPERO (ID: CRD42022308725). The authors report no conflicts of interest.

## Introduction

The advent of shape memory polymers (SMPs) has ushered in a new era in orthodontics, offering a blend of flexibility, adaptability, and responsiveness that is highly desirable in the fabrication of clear aligners. These smart materials possess the unique ability to return to a predetermined shape when exposed to specific stimuli such as heat or light, thereby enabling more controlled and potentially efficient tooth movement [1, 2]. As such, SMPs have garnered significant attention for their potential to revolutionize orthodontic treatment by offering programmable mechanical properties and extended activation capacity, while maintaining the advantages of conventional clear aligners in terms of aesthetics and patient comfort [3].

Chemically, these materials possess a dual-segment network which is a permanent ‘hard’ phase that defines the original shape (typically composed of crosslinked segments such as polyurethane (PU), polycaprolactone, or epoxy networks) and a ‘switching’ or *soft segment* that responds to external triggers like temperature, light, or moisture. When exposed to an external stimulus, the polymer chains transition from a temporary to a permanent configuration, enabling *shape recovery*.

In orthodontic applications, SMPs used in clear aligners are generally *thermoresponsive PU-based resins* or *methacrylate co-polymers* modified with aliphatic diisocyanates and poly(ε-caprolactone) segments. These materials exhibit glass transition temperatures (Tg) near intraoral conditions (35–45°C), allowing intraoral heat to activate gradual shape recovery and tooth movement. Some 3D-printable SMP resins, such as *Tera Harz TC-85* (Graphy, Korea), are urethane dimethacrylate (UDMA)-based photopolymers containing multifunctional acrylates and phosphine oxide photoinitiators. Their chemical design enables photopolymerization and thermo-mechanical memory, making them suitable for *4D-printing* of dental aligners.

This review therefore focuses specifically on PU- and methacrylate-based SMPs developed for orthodontic aligner fabrication, encompassing both *thermoformable* and *direct 3D-printed* variants [1–3].

Despite the advantages of conventional clear aligners, challenges such as frequent replacement, limited biodegradability, and concerns about material biocompatibility and sustainability remain important [4]. These materials have therefore been proposed as next-generation materials that may address these issues by offering extended activation capability and potential reductions in overall material waste.

This scoping review seeks to address these critical issues by mapping the current landscape of research on the health and environmental hazards associated with SMPs used in orthodontic aligners. By identifying existing evidence, highlighting research gaps, and outlining areas for future inquiry, this review aims to support the development of safer, more sustainable orthodontic materials and inform best practices in their use.

## Materials and methods

The protocol for this scoping review was registered on PROSPERO with ID CRD42022308725. The PRISMA-ScR checklist was followed, see Supplementary Material 1 [5]. Ethical approval was not required as this scoping review used exclusively anonymous information from publicly accessible documents.

### Research questions

The research question were (1) What are the environmental impacts associated with the production, use, and disposal of SMP in orthodontic aligners? (2) What are the potential health impacts associated using SMP in orthodontic aligners, including biocompatibility and potential adverse reactions? (3) What are the key research gaps in this area, and what future research is needed?

According to the PCC framework, the *population (P)*: The population includes studies related to SMP aligners across various study designs, including experimental studies (in vitro and in vivo), clinical trials, material degradation, and disposal methods. Only studies published in English within the last 10 years will be included. *concept (C)*: The focus is on the environmental and health implications of SMP aligners. This includes aspects such as waste generation, carbon footprint (environmental impact), biocompatibility, cytotoxicity, and potential allergic reactions (health impact). *context (C):* The review encompasses evidence from studies conducted globally to offer a comprehensive and international perspective on the topic.

### Search methods

A search strategy was designed with a combination of Medical Subject Headings (MeSH), title/abstract keywords, truncations, and Boolean operators. The search was performed on February 13, 2025, using the PubMed, Scopus, Embase, Web of Science, ProQuest, and Cochrane Library databases. Full searches of each database are provided in Supplementary Material 2–8.

To improve search sensitivity, additional free-text terms and synonyms were included, such as ‘shape-memory resin’, ‘3D-printed aligner resin’, ‘cytotoxicity of aligner resin’, and trade names like ‘Tera Harz’. This ensured retrieval of recent studies describing polymer leaching or resin-based SMP aligners not indexed under ‘shape memory polymer’.

Language restrictions were applied to English publications. The years of coverage of the databases spanned a period from 2015 to 12 February 2025.

### Selection criteria

In the first screening phase, titles and abstracts were viewed using Rayyan [6]. At least two reviewers (SA, MKN, SS), blinded to the other reviewer’s decision, assessed the inclusion or exclusion of articles based on the eligibility criteria. Disagreements were resolved through discussion. In the second screening phase, two reviewers (SA, MKN) assessed the full text of the articles for the final selection.

Inclusion criteria: Original studies (in vitro, in vivo, clinical, or environmental) investigating the biological, mechanical, or ecological effects of SMP-based orthodontic aligners were included. Eligible studies reported data on cytotoxicity, biocompatibility, mechanical properties, or environmental impact. Only full-text articles published in English within the past 10 years were considered.

Exclusion criteria: Studies were excluded if they evaluated conventional thermoformed aligners made from materials such as polyethylene terephthalate glycol (PETG), PU, or polycarbonate, which do not exhibit shape-memory behavior; review papers; case reports; editorials; abstracts without full data; and non-English publications.

The included study designs encompassed experimental studies (both in vitro and in vivo), clinical trials, material degradation assessments, and disposal-method evaluations. SMP-based aligners differ from conventional plastics in that they return to their original programmed configuration upon exposure to stimuli such as intraoral heat or light.

### Data collection and analysis

From the included studies, the key parameters were extracted from each study, stored in a Microsoft Excel spreadsheet, performed by SA, and then discussed with MKN to reach a consensus. The following parameters were extracted: Author(s), year of publication, study design, study location, sample size, polymer type, health hazards/ health risks identified, Environmental hazards/ impact, data quality, methodological approach, results & findings, conclusion/ recommendations (Table S1)

## Results

**Figure 1:**
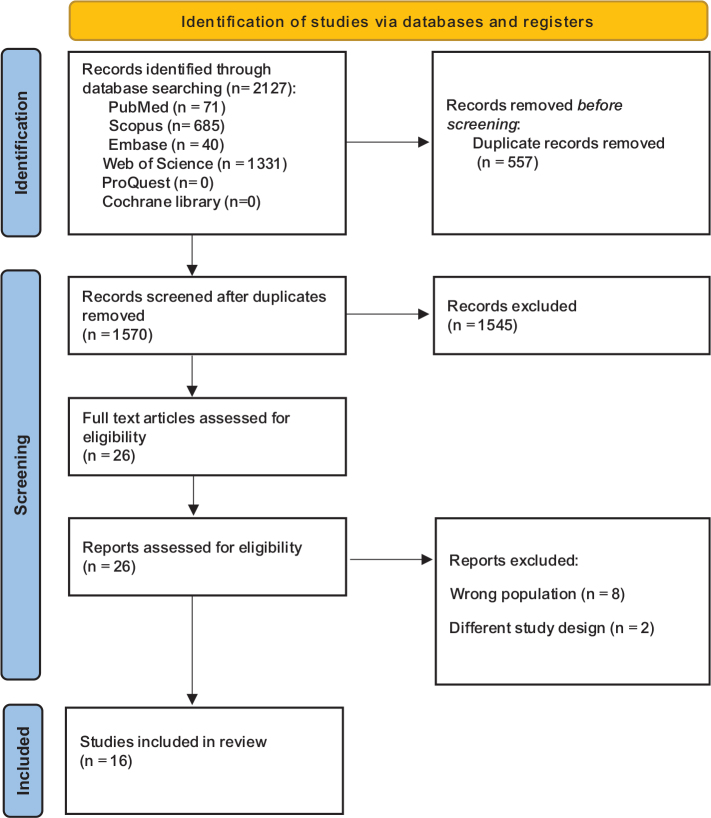
PRISMA flow diagram. Flow diagram of the final selection process.

As shown in the PRISMA diagram (Figure 1), a total of 1,570 unique articles were identified. After screening titles and abstracts, 1,545 studies were excluded. Full-text screening was conducted for the remaining 26 articles, of which eight were excluded due to an incorrect population and two due to an unsuitable study design. A total of 16 studies were included in the final analysis, as shown in Table S1, following the inclusion of an additional relevant study [14] that investigated UDMA leaching from 3D-printed aligner resins. Details of the excluded full-text studies, along with specific reasons for their exclusion, are provided in Supplementary Material 8 to enhance transparency and reproducibility.

## Discussion

The included studies collectively highlight evolving insights into the performance and safety profile of SMP-based orthodontic aligners. Several investigations explored the biocompatibility and cytotoxic risks associated with residual monomer release, emphasizing how polymer formulation and curing conditions influence cellular response and material behavior [1, 4, 2, 14]. In addition to biological considerations, thermo-mechanical responsiveness and activation efficiency were assessed to determine the stability of force delivery under intraoral conditions, demonstrating the potential of SMPs to maintain shape recovery and performance throughout treatment [3].

Environmental implications were also highlighted, including concerns related to polymer degradation, microplastic generation, and long-term waste management in clinical settings [3, 4]. Complementing these findings, conceptual and modeling advances have guided improvements in sustainable digital workflows and 4D-printing strategies aimed at enhancing the adaptability and efficiency of aligner manufacturing [17]. Collectively, this body of evidence demonstrates promising advancements in SMP-based aligner technology while reaffirming the need for continued research focused on long-term clinical performance, safety, and environmental impact.

Various studies have systematically evaluated the cytotoxic effects of materials employed in 3D-printed aligners. For instance, research conducted by [7] determined that there were no significant cytotoxicity or estrogenic effects associated with the Tera Harz TC85A resin. In contrast, a comprehensive literature review by Erbe et al. [8] emphasized that certain aligner materials release nanoplastics, which may pose substantial health risks, including cellular damage. The environmental implications of aligner production constitute a focal point of multiple studies. Notably, improper disposal of aligners contributes to microplastic pollution and an increase in non-biodegradable waste. A comparative life cycle assessment (LCA) conducted by Caelli et al. [9] demonstrated that the thermoforming process exhibits a higher environmental impact than 3D printing; this finding underscores the potential for industry shifts in manufacturing practices to alleviate ecological concerns [10].

Different polymer types show varied levels of biocompatibility and cytotoxicity, influenced significantly by curing processes and the presence of residual monomers. Numerous studies have indicated that photoinitiators, such as TPO (Diphenyl(2,4,6-trimethylbenzoyl)phosphine oxide) and BAPO (Bis(2,4,6-trimethylbenzoyl)phenylphosphine oxide), present health hazards due to their cytotoxic effects when not adequately cured [11]. The significance of post-curing processes is further highlighted by Campobasso et al. [12], who demonstrated that optimized post-processing protocols substantially reduce residual cytotoxicity. A separate study also reported that appropriate curing parameters and post-processing duration can further minimize the release of unreacted monomers, reinforcing the importance of standardized curing procedures in aligner fabrication [13]. These findings underscore the necessity of understanding how environmental factors within the oral cavity can affect material performance. Recent findings [7] detected measurable residual UDMA monomer release from a 3D-printed aligner resin and emphasized that optimized post-curing can markedly reduce cytotoxic potential [14].

Research exploring the integration of chitosan nanoparticles, as illustrated by Taher et al. [15], indicates that such modifications can enhance antibiofilm properties without compromising biocompatibility, thereby addressing both health and hygiene concerns. Furthermore, several studies employed rigorous methodologies compliant with international standards such as ISO 10993-5 for in vitro cytotoxicity and ISO 14040 for environmental assessment. For example, Elshazly et al. [1] and Atta et al. [4] conducted ISO-based cytotoxicity assays to evaluate polymer safety, while Lee et al. [2] applied standardized mechanical and thermal testing protocols to simulate intraoral conditions. Collectively, these investigations strengthen the reliability of biocompatibility and environmental evaluations across SMP and conventional polymer systems.

The present study emphasizes that unpolymerized acrylic resins may exhibit irritant and cytotoxic effects on cells due to the presence of residual monomers. These methacrylic monomers, if inadequately cured, have been shown to decrease the local pH and alter physiological properties, thereby contributing to potential adverse biological responses [16].

SmartTrack (PU) displayed the highest level of biocompatibility, maintaining cell viability of 94.07% ± 3.00, whereas Dental LT and E-Guard showed comparatively reduced cell viability values of 77.74% ± 3.22 and 75.06% ± 8.98, respectively [17].

The cleaning protocol following 3D printing plays a critical role in determining the surface characteristics and biocompatibility of clear aligners. Centrifugation proved effective in removing uncured resin residues immediately after printing. Increasing the centrifugation temperature to 55°C enhanced resin removal efficiency by reducing resin viscosity and improving flow dynamics. However, prolonged centrifugation (>2 min) at 55°C negatively affected aligner translucency, suggesting a thermal threshold for maintaining optical clarity [18].

Surface analysis revealed that the IPA-treated group exhibited the most irregular texture, whereas the NT group displayed a comparatively smoother surface. SEM evaluation further confirmed temperature-dependent morphological variations on the inner surfaces of the aligners. Importantly, variations in centrifugation temperature and duration did not significantly influence the shape memory properties of the materials, indicating structural stability under tested conditions. Inefficient resin removal, however, was associated with irregularities at the incisal edge, potentially leading to compromised fit and adaptation. Despite these differences, all cleaning protocols met ISO 10993-5 biocompatibility standards, confirming their suitability for intraoral application [18].

Pigmented materials, such as Pro Black, Med White, Biomed Black, and Biomed White, demonstrated a more pronounced reduction in mechanical performance compared to translucent materials like Med Amber and Biomed Amber. This disparity can be attributed to the pigment components that either absorb or reflect UV light, thereby hindering uniform light penetration and curing throughout the material [19].

The extent of biofilm formation is strongly influenced by material characteristics, including surface roughness, surface free energy, and chemical composition. Increased surface irregularities and higher surface energy promote bacterial attachment and biofilm maturation, whereas smoother and more hydrophobic surfaces tend to inhibit microbial colonization [20].

NextDent Ortho Flex demonstrated a higher storage modulus and glass transition temperature (Tg), indicating enhanced rigidity and superior thermal stability under elevated temperatures representative of intraoral conditions [21]. These properties suggest improved resistance to deformation and better mechanical performance during orthodontic loading. However, despite these mechanical advantages, NextDent Ortho Flex exhibited a relatively lower degree of conversion, raising potential concerns regarding the persistence of residual monomers after curing. In contrast, alternative formulations with reduced thermal stability may be more susceptible to phase transitions or deformation at physiological temperatures, thereby compromising their mechanical function and clinical reliability [21].

Increasing the thickness of direct-printed aligners (DPAs) was associated with a slight reduction in cell viability when cured according to the manufacturer’s recommended protocol of 20 min [22]. For thinner specimens (0.5 mm and 1 mm), cell viability decreased with longer curing times, whereas thicker specimens (2, 4, and 6 mm) exhibited the greatest reduction in viability at 30 min of curing, followed by a recovery at 60 min. This suggests that curing time interacts with material thickness to influence residual monomer release and cytotoxic potential. Additionally, exposure to saliva further reduced cell proliferation, highlighting the importance of intraoral environmental factors on material biocompatibility [22].

Polymethyl methacrylate (PMMA) was selected as the polymer of choice for microfluidic devices with biomedical applications due to its favorable print resolution, optical transparency, and biocompatibility [23]. In the present study, PMMA-based microfluidic chips demonstrated superior performance compared to traditional 96-well plate experiments for both cytotoxicity screening and osteogenic differentiation assays. The microfluidic platform offers the advantage of integrating all experimental steps – including preparation, treatment, cultivation, and analysis – on a single chip, thereby reducing variability and improving reproducibility of biological assays [23].

## Limitations

The research on dental aligners presents several limitations that warrant further consideration and investigation. One significant limitation is the variability in biocompatibility observed across different materials. Although some studies, such as those conducted by Pratsinis et al. [7], report no significant cytotoxicity for specific resins like Tera Harz TC85A, other research highlights cytotoxic effects associated with alternative photoinitiators such as TPO and BAPO when curing conditions are suboptimal [11]. This variability underscores the need for more comprehensive studies that systematically explore and compare the biocompatibility profiles of a wider range of materials under standardized testing conditions.

Another limitation is related to the scope of environmental assessments pertaining to aligner production. While studies such as the LCA by Caelli et al. [9] indicate that 3D printing may pose a lower environmental impact than traditional thermoforming techniques, the analysis is often confined to specific impact categories. This may overlook critical environmental considerations such as long-term ecological effects of microplastics released during production and disposal. Additionally, the studies frequently rely on modeling based on limited data sources, which can lead to conclusions that do not fully account for real-world practices and environmental impact variability.

Moreover, the research examining the effects of processing and post-curing techniques reveals significant gaps. Although some studies, including those by Campobasso et al. [12], emphasize the importance of effective post-processing to reduce toxicity, there is insufficient data on the optimal curing protocols necessary for different resin types or the long-term implications of these processes. This lack of standardized procedures hampers the ability to draw definitive conclusions about the safety and efficacy of various curing techniques.

Furthermore, the inclusion of nanoparticles, such as chitosan, in aligner materials raises questions about their overall impact on biocompatibility. While research, such as that by Taher et al. [15], suggests that the introduction of nanoparticles can enhance antibiofilm properties without significant cytotoxic effects, there is limited understanding of how different nanoparticle formulations interact with biological systems over extended periods. Future research must aim to clarify these interactions, emphasizing the need for both in vitro and in vivo studies to assess long-term safety.

Lastly, many studies rely heavily on controlled laboratory environments, which may not accurately reflect the complexities of the oral environment where dental aligners are used. Factors such as salivary composition, temperature fluctuations, and mechanical forces exerted during mastication can significantly influence material performance and cytotoxicity [4]. This limitation highlights the need for research designs that incorporate more ecologically valid models, preferably including longitudinal studies that consider how aligner materials perform in real-world conditions over time.

## Conclusion

While certain aligner materials exhibit acceptable biocompatibility, significant concerns regarding cytotoxicity arising from residual monomers persist. There is a critical need for ongoing monitoring and improved curing processes to mitigate potential health risks. Additionally, the environmental impact of aligner production can vary markedly between different manufacturing technologies, with advancements in 3D printing emerging as a promising alternative that could reduce carbon footprints while maintaining product efficacy. Future research should emphasize the exploration of long-term effects associated with microplastics and nanoplastics on human health, as well as the investigation of alternative polymer blends and optimization of production methods to enhance both biocompatibility and environmental sustainability. Clinicians are recommended to adhere strictly to manufacturer guidelines regarding processing and curing to minimize adverse effects during aligner treatment. Furthermore, patient education regarding material properties can serve to improve treatment outcomes. More broadly, discussions concerning material selection in both dental and medical fields should prioritize environmentally safe practices and advocate for a holistic approach that ensures the health of patients and the protection of the environment.

The current research has made valuable contributions to understanding the health and environmental impacts of dental aligners, substantial limitations remain.

## Supplementary Material


