# Supplementary material for: Health and environmental hazards of shape memory polymer used in orthodontic aligners – a scoping review
Source: Biomater Investig Dent. 2026 Jan 7;13:45225. doi: 10.2340/biid.v13.45225 (PMC12797256; doi:10.2340/biid.v13.45225)
Supplement: Supplementary file 1 [file BIiD-13-45225-s1.pdf]

S. NO	Author(s)	Year of Publication	Study Design	Study Location	Sample Size	Polymer Type
1	Pratsinis H etal	June, 2022	Lab based in-vitro study	Athens, Greece, Zurich, Switzerland, and Limassol, Cyprus	Ten sets of one type of aligner were used	Tera Harz TC85A aligner
	**Health Hazards/ health risks identified**	**Environmental Hazards/ impact**	**Data Quality**	**Methodological Approach**	**Results & Findings**	**Conclusion/ Recommendations**
	The study found no cytotoxicity to human gingival fibroblasts, no effects on intracellular ROS levels (oxidative stress), and no estrogenic effects from substances released by the 3D-printed aligners .	Not mentioned	cell culture assays and statistical analysis	laboratory analysis, specifically in vitro cell culture assays	Results showed no significant cytotoxicity or effects on ROS levels. Additionally, estrogen- sensitive MCF-7 and estrogen- insensitive MDA-MB-231 breast cancer cells were exposed to the eluates to assess estrogenicity, with no induction of MCF-7 cell proliferation observed.	Conclude that the specific resin used in this study for the 3D-printed aligner is biocompatible based on the assays performed . They suggest that analysis of the release of constituents or by-products of aligners is necessary to establish the first pool of evidence regarding their reactivity with aqueous media to derive information about potential release patterns in the oral cavity.
**S. NO**	**Author(s)**	**Year of Publication**	**Study Design**	**Study Location**	**Sample Size**	**Polymer Type**
2	Erbe C, Ludwig B, Bleilob M	2024	Literature review	Mainz, Germany	Not applicable	PET (Polyethylene Terephthalate)<br>PETG (Polyethylene Terephthalate Glycol)<br>TPU (Thermoplastic Polyurethane)<br>Graphy's resin (Tera Harz TC-85)
	Health Hazards/ health risks identified	Environmental Hazards/ impact	Data Quality	Methodological Approach	Results & Findings	Conclusion/ Recommendations
	Aligners release nanoplastics that can penetrate cell membranes,potentially causing cell destruction or mutation.Some aligner materials exhibit slight cytotoxicity, which can be amplified during the thermoforming process. An altered post-curing protocol can lead to moderate cytotoxicity. Prolonged UV curing may increase cytotoxicity. Severe allergic reactions requiring hospitalization have been reported.Variability in urethane dimethacrylate (UDMA) leakage from aligners may pose a potential health risk.Direct printed aligners (DPAs) can show increased surface roughness and porosity after intraoral use, raising hygiene concerns.Potential neurotoxic effects of micro and nanoplastics, including oxidative stress, neuronal damage, altered neurotransmitter levels,and behavioral changes.	Aligners contribute to microplastic pollution, with potential harm to ecosystems.Most aligners are made from non-biodegradable polymers, increasing environmental persistence.Waste generated from failed prints and post- processing adds to the environmental burden. Environmental concerns related to carbon emissions and the use of non-recyclable materials	Not applicable	Analysis of existing literature to assess the environmental and health impacts of aligners.<br>In vitro studies to evaluate the cytotoxicity and biocompatibility of aligner materials.Comparing the properties of different aligner materials and manufacturing methods	The orthodontic industry contributes to plastic pollution through the production and disposal of aligners. Certain chemicals released from aligners may pose health risks, including cytotoxicity and allergic reactions. The mechanical properties and biocompatibility of aligners vary depending on the material and manufacturing process.3D printing, also known as additive manufacturing, is now reaching a level of maturity	Lack of comprehensive data on the environmental impact of waste generated during the production of invisible aligners.<br>Some studies show good biocompatibility of certain materials (e.g., Graphy's Tera Harz TC-85) when used correctly, while others report cytotoxicity under altered conditions.<br>Need for more research on the long-term effects of microplastics and nanoplastics on human health and the environment
**S. NO**	**Author(s)**	**Year of Publication**	**Study Design**	**Study Location**	**Sample Size**	**Polymer Type**
3	Raszewski Z, Chojnacka K, Kulbacka J, Mikulewicz M	2023	laboratory experimental	Wroclaw Medical University and Wroclaw University of Science and Technology, Poland	Raw material composition included 120 samples for mechanical strength testing. For cytotoxicity assessment 15 samples were used. Additional tests involved 90 samples for sorption and solubility analyses.	Methacrylate, specifically: Ethoxylated (E4) bisphenol A dimethacrylate Urethane dimethacrylate (UDMA) Triethylene glycol dimethacrylate (TEGDMA) Pentaerythritol tetraacrylate
	**Health Hazards/ health risks identified**	**Environmental Hazards/ impact**	**Data Quality**	**Methodological Approach**	**Results & Findings**	**Conclusion/ Recommendations**

	The study assessed the potential for unpolymerized acrylic resins to be irritating and cytotoxic to cells, particularly related to residual monomer exposure that can lead to adverse health effects. The research highlighted the hazards linked to methacrylic monomers that could potentially decrease local pH and influence physiological properties if not adequately cured.	The transition from traditional methods to 3D printing can reduce waste generation, as fewer physical models and less material are required. Concerns were raised regarding the disposal of unpolymerized methacrylic compounds, which can be hazardous. The potential environmental impact of derived ions on local water systems was not specified but could be noted given the nature of ion release from the bioactive glass.	Study used standardized procedures and scientific methodologies, including UV-vis spectroscopy and precise measurements of released ions, to ensure the reliability and validity of the data. Sample preparation and testing conformed to established guidelines (e.g., ISO standards), indicating a high level of data quality.	Various bioactive glasses were mixed with methacrylate resins to create the 3D printing material. The samples were printed using a specific printer and post-cured under controlled conditions. Flexural strength was measured using three-point bending tests.Cytotoxicity was evaluated using human cell cultures, supplemented by the PrestoBlue assay.Ion release was quantified using plasma spectrometry at different pH levels and intervals (1, 28, and 42 days).	Samples with 10% Biomin C exhibited reasonable mechanical resistance, though less than traditional materials. The acrylic material showed high cell survival rates (over 85%) in human fibroblast tests, confirming good biocompatibility.Significant ion release (Ca^{2}?, PO?^{3}?, Si, and F?) was observed, correlating with increased bioactivity beneficial for dental health.	It concluded that the developed 3D printing material with bioactive glass has the potential for orthodontic applications due to its mechanical properties and bioactivity.<br>Recommendations for further research include: - Assessing different methacrylate compositions to enhance the material's mechanical resistance. - Exploring long-term clinical evaluations to confirm the functionality and safety of the material in clinical scenarios.
**S. NO**	**Author(s)**	**Year of Publication**	**Study Design**	**Study Location**	**Sample Size**	**Polymer Type**
**4**	Kim GT, Go HB, Yu JH, Yang SY, Kim KM, Choi SH, et al	2022	Laboratory-based investigation	Yonsei University College of Dentistry, Seoul 03722, Korea	Multiple groups with n = 3 for each resin test	BAPO (Benzoyl-Phosphine Oxide)<br>TPO (Diphenyl (2,4,6-trimethylbenzoyl) phosphine oxide) TPO-L (Ethyl (2,4,6-trimethylbenzoyl) phenylphosphinate)
	**Health Hazards/ health risks**	**Environmental Hazards/ impact**	**Data Quality**	**Methodological Approach**	**Results & Findings**	**Conclusion/ Recommendations**
	The cytotoxic effects were evaluated using the L-929 mouse fibroblast cell line, indicating that BAPO exhibited significantly higher cytotoxicity compared to TPO and TPO-L. Higher concentrations of BAPO and TPO increased cytotoxicity in cultured cells, raising concerns regarding their safety in clinical applications. Unreacted monomers and by-products of polymerization can diffuse into surrounding tissues, which may pose additional health risks.	The polymers used in dental applications may not be biodegradable, leading to potential long-term environmental contamination if disposed of improperly.Proper disposal methods for 3D printing materials and uncured resins are necessary to minimize environmental hazards.	The cytotoxicity tests followed ISO 10993-5: 2009 standards, ensuring the methodology is recognized and appropriate for biological eval uation. Use of statistical methods (ANOVA, Tukey's test) provides robustness to the findings, allowing for valid comparisons between groups. The tests were performed on multiple specimens (n = 3), enhancing the credibility of the results.	Standardized disc-shaped samples of resin were made for testing. Utilization of the MTT assay to evaluate cell viability after exposure to the resins and photoinitiators. Evaluation of color changes per ISO 4049: 2019 standards. Use of 3D scanning and analysis software for accuracy assessment. Three-point flexural strength and hardness tests measured using controlled protocols.	The TPO-L exhibited the highest cell viability (89.62 ± 4.93%), while BAPO showed the lowest (74.16 ± 3.7%). BAPO showed significant discoloration (?E > 3.3), whereas TPO and TPO-L showed better color stability. The TPO-L group demonstrated superior dimensional accuracy with less deviation in the Z-axis and XY-axis compared to BAPO. All groups exhibited similar mechanical characteristics; however, the absolute values were below the desirable threshold set by ISO standards.	The TPO-L photoinitiator shows promise for clinical use due to lower cytotoxicity and better performance in color stability and dimensional accuracy. Given the health risks associated with BAPO and TPO, it is advisable to limit their use or consider alternative formulations. Further studies on the long-term effects of residual monomers, optimization of curing procedures, and potentially the introduction of fillers to improve mechanical properties are necessary to enhance the application of 3D printing resins in dentistry.
**S. NO**	**Author(s)**	**Year of Publication**	**Study Design**	**Study Location**	**Sample Size**	**Polymer Type**
**5**	Campobasso A, Ariano A, Battista G, Posa F, Migliorati M, Drago S et al	July, 2023	In vitro experimental study	University of Foggia, University of Genova, University of Ferrara, Italy	Six aligners were used for each post-curing procedure (P1 and P2)	TC-85DAC (Graphy, Seoul, Korea)
	**Health Hazards/ health risks identified**	**Environment al Hazards/ impact**	**Data Quality**	**Methodological Approach**	**Results & Findings**	**Conclusion/ Recommendations**

	Uncured 3D-print resins are highly cytotoxic. Post-polymerization is necessary to remove uncured resin and reduce toxicity . The cytotoxicity of 3D-print materials can be affected by material composition, printing conditions, and post-processing procedures . Each step in the material's processing stage may negatively impact its biocompatibility .Incomplete surface polymerization can result in an under-cured polymer with unreacted oligomers and monomers. Minimizing residual monomer content in 3D-printed aligners is necessary to reduce later contact with oral tissues .	Not mentioned	The study uses optical density (OD) measurements from the MTT assay to quantify cell viability .Two-way ANOVA and one-way ANOVA with Tukey's post hoc test were performed to analyze the differences between groups . The level of significance was set at P ≤ 0.05 . The study includes positive (cells and medium) and negative (medium only) controls to account for background interference .Two independent experiments were performed after 7 and 14 days, each with four triplicates.	MC3T3E-1 mouse pre-osteoblasts were cultured in DMEM supplemented with fetal bovine serum and antibiotics .Sample Aligners were 3D-printed using TC-85 resin and post-cured under two different conditions (P1 and P2) .The MTT assay was used to assess cell viability. Cells were treated with MTT solution, and the formazan was solubilized using DMSO .The optical density (OD) of each well was measured using a spectrophotometer at 595 nm .Data were analyzed using two-way ANOVA and one-way ANOVA with Tukey's post hoc test.	Statistically significant differences between the groups and MTT timings were demonstrated using two-way ANOVA (P < 0.001) .The P2 group exhibited significantly lower values compared to the P1 and C+ groups (P < 0.001) .The P1 group showed high cyto-compatibility after 7 and 14 days, with cell viability around 107% .The P2 procedures reported significantly moderate cytotoxicity (P < 0.001), with a mean percentage of surviving cells of 59.79% after 7 days and 47.09% after 14 days .The study suggests that the increased light intensity and nitrogen environment of the THC-2 device (P1) improved cell viability, while the less powerful UV lamp and lack of oxygen inhibition with the FormCure machine (P2) resulted in moderate cytotoxicity.	Different post-polymerization procedures may affect the in-vitro cytotoxicity of 3D-print resin .3D-printed aligners post-cured using a Tera Harz Cure incorporating a nitrogen generator were found to be biocompatible .Post-cured using FormCure resulted in mild cytotoxicity .Orthodontic laboratories and clinicians should follow the manufacturer's recommendations to avoid possible toxic effects during aligner treatment.
**S. NO**	**Author(s)**	**Year of Publication**	**Study Design**	**Study Location**	**Sample Size**	**Polymer Type**
6	Ahamed SF, Kumar SM, kumar V, Kanna A, K Indrapriya dharshini	2020	A laboratory-based in vitro prospective cytotoxicity study.	Sri Ramakrishna Dental College and Hospital, Tamil Nadu, Coimbatore, India	Each set involving multiple wells (e.g., 28 wells per material per extraction time point) plus a control group of 7 wells. The repeated measurements over four time intervals (day 1, 3, 5, and 7) ensured a robust data set for evaluating time-dependent cytotoxicity.	Two types of 3D-printed photopolymer resins-Dental LT and E-Guard clear-used directly for aligner fabrication, and a thermoformed polyurethane material (SmartTrack) used in Invisalign aligners
	**Health Hazards/ health risks identified**	**Environment al Hazards/ impact**	**Data Quality**	**Methodological Approach**	**Results & Findings**	**Conclusion/ Recommendations**
	All materials exhibited cytotoxicity to some degree, with the direct-printed resins (Dental LT and E-Guard clear) showing slight cytotoxic effects due to the leaching of methacrylate monomers during initial usage, which can affect cell viability. SmartTrack (polyurethane) was found to be the most biocompatible with cell viability as high as 94.07% ± 3.00, while Dental LT and E-Guard showed lower viability (77.74% ± 3.22 and 75.06% ± 8.98, respectively)	Safe postprocessing (washing and post-curing) minimizes uncured resin release, indirectly reducing environmental contamination during clinical use and disposal.	In vitro prospective cytotoxicity experiment using standardized methods (MTT assay) and ISO protocols for specimen preparation and extraction. Multiple time-point assessments (days 1, 3, 5, and 7) and statistical analysis performed via SPSS with ANOVA and Bonferroni post-hoc tests ensure that the data are statistically robust, even though sample size per group (n = 7 wells per time interval) remains a typical limitation in cell-based assays.	In vitro design using 3T3 embryonic mouse fibroblast cell lines to assess the cytotoxicity of both directly 3D-printed and thermoformed aligners via the MTT assay. Extraction of aligner samples was performed by measuring specimen surface areas, immersing them in Dulbecco's Modified Eagle Medium at prescribed intervals (days 1, 3, 5, and 7), and then assessing cell viability using a standardized optical density measurement at 540 nm after incubating with the MTT reagent and DMSO dissolution. Statistical significance was evaluated with one-way ANOVA and Bonferroni post-hoc tests at a p-value threshold of 0.05.	All tested materials exhibited slight cytotoxicity, with the highest toxicity observed on day 1; toxicity levels decreased significantly by day 7, reflecting initial leaching of cytotoxic components that subsided over time. Statistically, Invisalign (SmartTrack) demonstrated superior cell viability (94.07% ± 3.00), while both Dental LT and E-Guard clear direct-printed aligners maintained lower viability rates in the range of 75–78% . There was a significant difference in cell viability between the thermoformed and 3D-printed groups but no significant difference between Dental LT and E-Guard, indicating similar performance for the direct-printed materials	Direct-printed aligners (Dental LT and E-Guard clear) exhibit slight cytotoxicity, their biocompatibility is within acceptable limits compared to conventional thermoformed aligners. SmartTrack (polyurethane) remains the most biocompatible option amongst the materials tested, suggesting that its use in aligner fabrication results in fewer adverse biological effects. It is recommended to refine post-processing protocols (post-curing and washing) for 3D-printed materials to reduce residual monomer content and further improve biocompatibility. Future research should explore alternative materials (such as novel EnvisionTEC products) and extend cytotoxicity assessments beyond a one-week period, ideally using multiple cytotoxicity assays to fully validate safety for long-term intraoral use .
**S. NO**	**Author(s)**	**Year of Publication**	**Study Design**	**Study Location**	**Sample Size**	**Polymer Type**
7	Taher, B.B.; Rasheed, T.A	2023	An in vitro investigation	University of Sulaimani, Iraq	There were ten samples per group: G1, Thr; G2, CR; G3, CR + 2% Chs; G4, CR + 3% Chs; and G5, CR + 5% Chs	Dental LT clear resin was used, modified with chitosan nanoparticles (83.5% deacetylation, 74 nm, 50- 190 kDa) to enhance antibiofilm efficacy while maintaining acceptable mechanical properties.
	**Health Hazards/ health risks identified**	**Environmental Hazards/ impact**	**Data Quality**	**Methodological Approach**	**Results & Findings**	**Conclusion/ Recommendations**

	Cytotoxic potential of the 3D-printed aligner material (Dental LT clear resin, CR) and found that its inherent cytotoxicity was low. With the incorporation of chitosan (Chs) nanoparticles at different percentages (2%, 3%, and 5%), the cell viability remained in acceptable ranges. According to ISO 10993 guidelines, cell viabilities above 90% indicate no cytotoxic effects, while those between 60- 90% imply minimal toxicity. Although chitosan offers antibiofilm benefits, the study also notes that the cytotoxic profile of Chs depends on factors such as particle size, degree of deacetylation, and molecular weight. However, the inserts of Chs did not compromise the resin's biocompatibility.By reducing bacterial adhesion (notably against Streptococcus mutans), the modified aligners help decrease the health risks associated with dental caries and periodontal diseases.	Although the study doesn't deeply address environmental release, the inclusion of nanoparticles (Chs) brings up general environmental concerns about nanoparticle release; however, their usage here is controlled, and the benefits (improved antibiofilm properties) may offset risks associated with increased material durability.	Comparative statistical analyses (e.g., confidence intervals, descriptive tables) that contribute to the robustness of the findings. The inclusion of multiple measures (antibiofilm, mechanical tests, degree of conversion, and cytotoxicity assays) enhances data triangulation, indicating good quality and reliability of the data collected.	FTIR-ATR was used to evaluate the degree of polymer conversion, which highlights unpolymerized material and the influence of nanoparticle inclusion. Cytotoxicity was evaluated using cell culture methods (ISO 10993 guidelines) and antibiofilm activity was assessed by measuring colony-forming units after exposing the material to cariogenic bacteria.	Adding 3% and 5% Chs to the resin led to a significant reduction in bacterial colonies (31% and 70%, respectively) compared to thermoformed aligners (Thr).	Incorporating chitosan nanoparticles into the 3D-printed Dental LT clear resin enhances antibiofilm activity, reducing the adhesion of Streptococcus mutans, a key cariogenic bacterium, without compromising the resin's mechanical and biological properties. Further testing, especially in vivo studies on human subjects, is recommended to validate these in vitro findings. Future research could explore varying concentrations of Chs and the inclusion of other nanoparticles to potentially further optimize clinical performance.
**S. NO**	**Author(s)**	**Year of Publication**	**Study Design**	**Study Location**	**Sample Size**	**Polymer Type**
**8**	Kim JE, Mangal U, Yu JH, Kim GT, Kim H, Seo JY, et al.	2024	Experimental study investigating the effects of centrifugation cleaning methods on 3D-printed clear aligners, with a focus on surface characteristics and optical features.	Yonsei University College of Dentistry, Seoul, Republic of Korea	The study involves multiple groups of clear aligner specimens (NT, IPA, RT-2, RT-4, RT-6, HT-2, HT-4, HT- 6) to evaluate the impact of different cleaning methods.	Specific type of resin used for 3D printing the clear aligners is not explicitly stated in this excerpt.
	**Health Hazards/ health risks identified**	**Environmental**	**Data Quality**	**Methodological Approach**	**Results & Findings**	**Conclusion/**
	The study found that all cleaning methods complied with ISO 10993-5 standards, suggesting that the aligners are biocompatible for oral use regardless of the cleaning method. The NT (not treated) group showed the lowest cell survival rate, indicating that residual uncured resin can pose a cytotoxic risk. Effective cleaning methods are essential to mitigate this risk.	The study mentions the use of an organic solvent (isopropyl alcohol, IPA) and highlights an organic- solvent-free centrifugation method as an alternative for cleaning, potentially reducing environmental hazards associated with solvent disposal.The use of 3D printing in aligner production, with its ease of production, could lead to environmental benefits through optimized material usage.	The study presents quantitative data for shape recovery ratio (Table 1), RMS values, and UV- vis transmittance. Qualitative assessments were performed using SEM images and color- difference maps. Statistical significance was assessed for RMS values and shape recovery ratios, enhancing the reliability of the findings.	Clear aligners were 3D-printed and subjected to different cleaning methods: centrifugation at room temperature (RT) and high temperature (HT) for 2, 4, and 6 minutes, isopropyl alcohol (IPA) cleaning, and no treatment (NT). SEM images were used to analyze the surface morphology of the aligners. - UV- vis transmittance curves were used to evaluate the transparency of the aligners. Color-difference maps and RMS values were used to compare the effect of inadequate uncured resin removal on the fit of clear aligners.The shape recovery ratio was measured to assess the impact of cleaning methods on the aligners' mechanical properties. Cell viability assays were performed to assess the cytotoxicity of the aligners after different cleaning methods.	Centrifugation is effective for removing uncured resin from clear aligners immediately after 3D printing.Increasing the temperature to 55 °C during centrifugation enhanced resin removal by reducing its viscosity and improving flow. Centrifugation at 55 °C for more than 2 minutes negatively impacted the translucency of the aligners. The IPA group had the most irregular surface texture, while the NT group's surface was considerably smoother. SEM revealed noticeable differences in the inner surface of the aligners with increasing temperature. The centrifugation cleaning temperature and time did not significantly compromise the shape memory properties of clear aligners. Inefficient resin removal adversely affects the incisor edge, which can lead to unintentional fit issues. All groups complied with ISO 10993-5 standards, indicating biocompatibility for oral use regardless of the cleaning method.	Centrifugal cleaning at 55 °C for 2 minutes with a force of 27.95 g effectively removes uncured resin from aligners while maintaining clinically desirable esthetics. The study recommends the use of centrifugation cleaning at 55°C for 2 minutes to effectively remove uncured resin. Limitations of the Study: Iincludes the lack of standardized indicators for transparency and the use of aligners produced from a standardized typodont model.
**S. NO**	**Author(s)**	**Year of Publication**	**Study Design**	**Study Location**	**Sample Size**	**Polymer Type**

9	Guttridge C, Shannon A, O'Sullivan A, O'Sullivan KJ, O'Sullivan LW	May, 2024	Experimental study	University of Limerick, Ireland	Two different vat-polymerization 3D printer ranges, with samples created in concentric layers to assess curing effectiveness. Sample sizes and replicates per group are detailed in Table 2 for UTS, Young's Modulus and Elongation at break, across the materials and layers tested.	Translucent materials (Med Amber, Biomed Amber) and pigmented materials (Pro Black, Med White, Biomed Black, Biomed White).
	**Health Hazards/ health risks identified**	**Environmenta l Hazards/ impact**	**Data Quality**	**Methodological Approach**	**Results & Findings**	**Conclusion/ Recommendations**
	The presence of uncured resins in 3D-printed parts, even after post- processing, is a significant concern due to potential toxicological and immunological responses. Studies have identified leachates of uncured resin from printed models, which further post-processing does not entirely eradicate, posing biocompatibility concerns. Reduced mechanical properties imply the presence of incompletely cured resins, which may negatively impact the biocompatibility of the finished part.	Not mentioned	The study presents data for ultimate tensile strength (UTS), Young's modulus (YM), and elongation at break (EB) for each material and layer (Table 2).	Samples were post-cured at two intervals (100% and 500% of the manufacturer's recommended time) to assess the impact of curing duration. Ultimate tensile strength (UTS), Young's modulus (YM), and elongation at break (EB) were measured to evaluate mechanical properties. Samples were analyzed in concentric layers (A, B, C, D) to evaluate the effects of geometry on curing effectiveness.	Mechanical properties decreased from outer layer A to inner layer D, indicating that internal features affect post-processing effectiveness. Pigmented materials (Pro Black, Med White, Biomed Black, Biomed White) showed a greater reduction in mechanical properties compared to translucent materials (Med Amber, Biomed Amber), suggesting that pigmentation absorbs and/or reflects UV light. Extending post-curing times to 500% did not result in homogenous properties throughout the parts, especially for pigmented resins. The reduced mechanical properties imply the presence of incompletely cured resins, raising concerns about the biocompatibility of complex geometry 3D-printed parts.	Caution must be taken when post-curing parts printed on vat-polymerization 3D printers, as generic instructions may not result in sufficient curing of the entire part if the model is complex in design. Translucent materials appear to perform best when creating large parts or those with complex internal geometries. Future research should consider alternative materials and different complexity of designs of print models. Future studies should also consider assessing the biocompatibility after post processing, including potentially in vitro cytotoxicity assessment in accordance with ISO 10993 to correlate the relationship between residual monomers with mechanical properties.
**S. NO**	**Author(s)**	**Year of Publication**	**Study Design**	**Study Location**	**Sample Size**	**Polymer Type**
10	Moradinezhad et al.	2024	in vitro experimental study	Ahvaz, Iran	345 specimens (270 test discs and 45 negative controls)	3D printed CAD/CAM material (Detax) and four thermoformed retainers [Erkodent (polyethylene terephthalate glycol [PETG]); EasyVac (polyethylene); DB (polyester based on terephthalic acid); and Clear Tech]
	**Health Hazards/ health risks identified**	**Environmenta l Hazards/ impact**	**Data Quality**	**Methodological Approach**	**Results & Findings**	**Conclusion/ Recommendations**
	The study investigates biofilm formation, which is a risk factor for oral diseases. Microbial adhesion to the surfaces of retainers can lead to biofilm formation and subsequent health issues. Surface roughness, surface free energy, and chemical composition of the materials can impact biofilm development.	Not mentioned	The study uses optical density (OD) measurements to quantify biofilm formation. Two-way ANOVA and Tukey tests were used to analyze the data, with a significance level set at $\alpha = 0.008$	Clear retainers of different brands/materials were prepared according to the usual manufacturing process. Retainers were exposed to different microorganisms over different time intervals (24 h, 72 h, 120 h). Biofilm formation was quantified by measuring the optical density (OD) of the samples.	No significant overall difference was observed among the clear retainer materials tested in terms of biofilm formation. A significant overall increase in biofilm formation was observed between the first and third days, with no significant increase between days 3 and 5. Candida albicans biofilm was more intense than the tested 5 bacteria.	As far as the six microorganisms tested are concerned, there might not be a significant overall difference among the clear retainer materials. Clinical studies are needed to verify the results. The authors suggest future research should focus on longer durations and more closely mimic clinical conditions to enhance result validity.
**S. NO**	**Author(s)**	**Year of Publication**	**Study Design**	**Study Location**	**Sample Size**	**Polymer Type**
11	Simunovic L, Maric AJ, Bacic I, Haramina T, Mestrovic S.	2024	In vitro experimental study	University of Zagreb,Croatia	Exposure times of 5 s, 6 s, 7 s, and 8 s for NextDent and 2 s, 2.4 s, 3 s, 4 s, and 4.5 s for Tera Harz were used. For each group, 10 samples were prepared (in total, 90 samples).	Tera Harz TC-85 resin (Graphy, Seoul, Republic of Korea) and NextDent Ortho Flex (Vertex-Dental B.V., Soesterberg, The Netherlands)
	**Health Hazards/ health risks identified**	**Environmental Hazards/ impact**	**Data Quality**	**Methodological Approach**	**Results & Findings**	**Conclusion/ Recommendations**

	A lower degree of conversion (DC) in some formulations raises concerns about residual monomers, which can be cytotoxic or cause adverse tissue reactions when in contact with oral tissues Materials that become less stable at mouth temperature could potentially deform, leading to failures in orthodontic forces and unintended contact with soft tissues.	Although not the primary focus, reduced DC and residual monomers may also have implications for environmental disposal and biocompatibility of waste materials.	High-quality data acquisition is implied through the measurement of Tg (glass transition temperature), and degree of conversion. he inclusion of multiple replicates and appropriate statistical tests (likely ANOVA or t-tests) ensures that differences between resin formulations are significant and reproducible.	Different post-curing conditions are applied to the resin specimens to assess how curing parameters affect the degree of conversion and subsequent performance. Methods such as Fourier-transform infrared spectroscopy (FTIR) may be used to quantify the degree of conversion and detect residual monomers. The comparative design allows for direct assessment of how modifications in the curing process can enhance or compromise material properties and clinical safety.	NextDent Orto Flex demonstrated a higher storage modulus and Tg, providing greater rigidity and thermal stability at elevated temperatures typical of the oral environment. Despite its mechanical advantages, NextDent Orto Flex exhibited a lower degree of conversion, raising concerns about residual monomers persisting after curing. Alternative formulations with lower thermal stability risk phase changes or deformation at mouth temperature, compromising their function under orthodontic forces. Data supports the conclusion that customized post-curing protocols are needed to balance the benefits of rigidity and thermal stability with complete polymerization to minimize health risks.	The study underscores the importance of optimizing post-curing protocols to maximize the degree of conversion without sacrificing thermal and mechanical performance. Clinicians must consider both the thermal properties and curing efficiency of dental resins to ensure long- term stability and biocompatibility in orthodontic applications. Emphasis is placed on reducing residual monomers to mitigate potential health risks. Further investigations are recommended to refine curing parameters and explore new resin formulations that offer both high thermal stability and complete polymerization, ensuring they meet clinical safety standards while delivering optimal performance under orthodontic forces.
**S. NO**	**Author(s)**	**Year of Publication**	**Study Design**	**Study Location**	**Sample Size**	**Polymer Type**
**12**	Iodice G, Ludwig B, Polishchuk E, Petruzzelli R, Di Cunto R, Soboh Husam, et al.	May, 2024	In vitro experimental study	Italy, Sweden, Romania, New Zealand	a total of 60 square samples (10 mm × 10 mm; thickness 0.5 mm) of aligner material were directly 3D printed using Tera Harz TC-85 DAC resin (Grapy, Seoul, South Korea) For the control group, 20 flat square samples (10 mm × 10 mm; thickness 0.76 mm) of Zendura FLX (Zendura Dental, Fremont, United States) were utilized.	Tera Harz TC-85 DAC resin and zendura FLX
	**Health Hazards/ health risks identified**	**Environmental Hazards/ impact**	**Data Quality**	**Methodological Approach**	**Results & Findings**	**Conclusion/ Recommendations**
	The study demonstrated that all 3D-printed aligner samples exhibited a significant cytotoxic effect on human fibroblast growth compared to a sterile glass control, indicating potential risks to oral soft tissues .A prolonged curing time specifically the 50-minute group was associated with a significantly higher reduction in cell growth (approximately 39.3 cells per field), suggesting that overcuring may exacerbate cytotoxic effects .The findings also highlighted that pure saliva reduced cell viability, which implies that the oral environment with its enzymatic components could further influence the toxicity profile of these materials.	The research compared the cytotoxic impact of aligner eluates immersed in DMEM culture medium versus centrifuged human saliva, revealing differences in cell viability that may be relevant to how the aligners perform in the variable oral environment . This suggests an environmental impact where factors such as the presence of salivary enzymes and other physiochemical conditions could affect not only the material's degradation but also its interaction with surrounding tissues.	The results are based on two independent experiments performed in duplicate, which provides a measure of reliability and consistency despite inherent in vitro limitations .Statistical analyses revealed highly significant differences ($p < .001$) when comparing various curing times and control groups, establishing a clear negative linear correlation between curing time and fibroblast growth .However, the study acknowledged that in vitro models may not fully replicate the complexity of the oral environment, thus highlighting a need for cautious extrapolation of the data to clinical scenarios.	The study employed an immunofluorescence test to quantify fibroblast growth on aligner surfaces, comparing different curing time protocols (14, 24, and 50 minutes) against a glass control and a conventional thermoformed aligner (Zendura FLX) group . In addition, an MTT cell viability assay was used to assess fibroblast survival when aligners were immersed in both DMEM and centrifuged saliva over a 14-day period, thereby simulating different in vitro conditions .	All groups of 3D-printed aligners showed a significant reduction in fibroblast growth compared to the glass control, with the 50-minute curing time group exhibiting the most pronounced cytotoxic effect .A negative linear trend was identified, indicating that increased curing time correlates with greater cytotoxicity, even though no significant difference in fibroblast viability was observed among the shorter curing time groups .The study also found that the medium used (saliva versus DMEM) influenced cell viability; fibroblasts in saliva showed significant reduction, likely due to enzymatic effects, while the difference in DMEM was less pronounced though still present .	Directly printed aligners show a cytotoxicity profile similar to conventional thermoformed aligners in terms of fibroblast growth, but the level of cytotoxicity increases with extended curing times, reinforcing that prolonged post-curing can be detrimental . The study recommends strict adherence to the prescribed curing protocols (e.g., shorter durations such as 14 or 24 minutes), as over-curing (50 minutes) appears to worsen the cytotoxic effect possibly due to alterations in surface characteristics like increased roughness or porosity .Future research is advised to explore the underlying mechanisms such as the role of temperature, material porosity, and residual stress that lead to increased cytotoxicity, as well as to conduct in vivo studies that capture additional variables such as chewing forces, thermal changes, and chemical exposures in the oral environment
**S. NO**	**Author(s)**	**Year of Publicat ion**	**Study Design**	**Study Location**	**Sample Size**	**Polymer Type**
**13**	Bleilöb M, Welte-Jzyk C, Knode V, Ludwig B, Erbe C.	2025	In-vitro study	University Mainz, Germany. University of Homburg, Saar, Germany.	Not applicable	Graphy's aligner resin, Tera Harz TA-28
	**Health Hazards/ health risks identified**	**Environ mental Hazards / impact**	**Data Quality**	**Methodological Approach**	**Results & Findings**	**Conclusion/ Recommendations**

	Potential exposure to cytotoxic monomers due to insufficient UV curing, especially in areas with higher material thickness . Oral mucosa irritation has already been detected in some patients. UDMA leaching from DPAs made from Tera Harz TC-85 . Increased cytotoxicity with prolonged curing durations .	Not mentioned	The study highlights the high accuracy of the production method, with all specimens achieving their target dimensions regardless of UV curing time.	Evaluated the biocompatibility of DPAs made from Tera Harz TA-28 . Assessed the effects of different material thicknesses on cell viability .Analyzed the effects of eluates from specimens of different thicknesses, cured for various durations (20, 30, or 60 min), on HGFs . Incubated samples in saliva to observe the impact on cell proliferation.	Increasing thickness of DPAs resulted in slight loss of cell vitality when following company's curing guidelines of 20 minutes .For specimens with 0.5 mm and 1 mm thickness, cell viability decreased with increasing curing time .Specimens with 2, 4, or 6 mm thickness showed the greatest loss in cell viability at a curing time of 30 min, with viability increasing again at 60 min . Saliva exposure significantly reduced cell proliferation. The current study confirmed the high accuracy of the production method, as all specimens achieved their target dimensions irrespective of the UV curing time.	The standard 20-minute UV curing protocol is sufficient to ensure biocompatibility and patient safety for material thicknesses up to 6 mm . The study challenges Graphy's recommendation to extend UV curing by an additional 10 min for DPAs made from TC-85 that are thicker than 0.7 mm . It is recommended not to increase UV curing duration for layer thicknesses of up to 6 mm to optimize time and enhance productivity . The study emphasizes the necessity of considering intraoral factors such as salivary enzymes, pH variations and microbial interactions, which may alter material biocompatibility.
**S. NO**	**Author(s)**	**Year of Publicat ion**	**Study Design**	**Study Location**	**Sample Size**	**Polymer Type**
**14**	Riester O, Laufer S, Deigner HP.	2022	Experimental study	Furtwangen University, Germany	Not explicitly stated, but the study involves multiple experiments with cell cultures, microfluidic chips, and polymer discs	PLA-transparent (PLA300XCLR) . PMMA-transparent (PMM300XCLR). PC-transparent (PCA300XCLR).
	**Health Hazards/ health risks identified**	**Environ mental Hazards / impact**	**Data Quality**	**Methodological Approach**	**Results & Findings**	**Conclusion/ Recommendations**
	The study primarily focuses on biocompatibility, so direct health hazard identification is limited. Cytotoxicity of staurosporine was assessed using microfluidic chips.<br>The study aims to find biocompatible materials for microfluidic devices used in cell culture, reducing potential risks associated with less biocompatible materials .	Not mentioned	Resolution assessment of 3D printed microfluidic channels was performed by comparing achieved channel widths and heights with CAD model values. Theoretical fluid compositions at the outlets of the microfluidic gradient generator were verified by injecting red and blue solutions and measuring absorbance .<br>Biocompatibility analyses were performed with MTT assay to assess cell viability.	Fabrication of microfluidic chips using an Ultimaker 3 FDM 3D printer . Resolution assessment of direct 3D printed microfluidic channels . Testing and characterization of microfluidic devices with different chip components . Microfluidic gradient generator was used tocreate concentration gradients . Biocompatibility analysis of chip materials using MTT assay . Microfluidic cytotoxicity assay to determine the GI50 of staurosporine . Microfluidic ALP activity assay to assess osteogenic differentiation . Numeric simulation of fluidic behavior in microfluidic chips using FEM.	PMMA was selected as the polymer of choice for microfluidic devices with biomedical applications based on print resolution, optical transparency, and biocompatibility . Microfluidic chips showed better performance than 96- well plate experiments for both cytotoxic screening and osteogenic differentiation. The microfluidic system allows all steps of an experiment (preparation/treatment, cultivation, analysis) to be performed on one chip.	Microfluidic methods offer many advantages over current standard methods, especially in dynamic cell culture systems .3D printing is suitable for reproducible production of microfluidic channels and structures .Self-printed microfluidic chips are applicable and superior in cell culture, cytotoxicity assessment, and stem cell differentiation analysis . The chips can be easily adapted to specific experimental designs by modifying the number of cell culture chambers, integrating sensor systems, adding new inlets, or using different chip materials.
**S. NO**	**Author(s)**	**Year of Publication**	**Study Design**	**Study Location**	**Sample Size**	**Polymer Type**
**15**	Caelli C, Tamburrino F, Brondi C, Razionale AV, Ballarino A, Barone S	2023	Comparative Life Cycle Assessment (LCA)	Milan, Italy, Pisa, Italy	The functional unit is a complete set of 40 aligners	Polyethylene terephthalate glycol (PETG) copolymer (for thermoforming) 1. Aliphatic vinyl ester-urethane polymer, possibly cross-linked with methacrylate functionalization (for direct printing)
	**Health Hazards/ health risks identified**	**Environmental Hazards/ impact**	**Data Quality**	**Methodological Approach**	**Results & Findings**	**Conclusion/ Recommendations**

	The study does not focus on direct health hazards, but rather on the environmental impacts of the materials and processes . The mention of material safety data sheets suggests awareness of potential hazards associated with the materials.	The study assesses potential environmental impacts using the EF 3.1 method across 25 impact categories. Key impact categories analyzed include climate change, energy resources, acidification, and eutrophication. Thermoforming has a higher potential impact for all impact categories considered compared to direct 3D printing . Raw materials are the main impact driver for thermoforming, with energy and waste also contributing significantly.Water use is significantly different between the two technologies due to the washing of soluble supports in thermoforming.	Data was obtained from the Ecoinvent database and safety material data sheets . Modelling of materials involved simulating synthesis routes based on literature due to the unavailability of precise material data .The study acknowledges limitations related to the lack of specific compound information in material safety data sheets and the patented composition of the direct printing material .	Comparative LCA of thermoforming and direct 3D printing (DLP) for dental aligner production. Cradle-to-gate analysis encompassing raw material extraction, processing, aligner production and packaging.Use of the EF 3.1 impact method to assess potential environmental impacts across various categories. Modelling of polymeric materials based on existing sheets in the Ecoinvent database and safety material data sheets. Assessment of energy consumption based on the Italian residual mix and transport data from market- type databases.	Thermoforming has a higher potential environmental impact than direct 3D printing across all impact categories considered. The smallest gap between the two technologies occurs in the "Land use" category (31% change), and the largest gap occurs in the "Ozone depletion" category (98% reduction in impact). The reduction in environmental impact for direct printing is primarily due to the decrease in the quantity of material and energy used.	Direct printing of dental aligners has a lower environmental impact than thermoforming . This advantage is primarily attributed to the efficient use of materials, reduced energy consumption, and minimized waste generation associated with DLP. The study suggests extending the application of environmental effectiveness to other possible areas in the biomedical field and comparing it with different technologies.Future research should include an update of existing databases and greater disclosure of material composition, overcoming patent barriers.
**S. NO**	**Author(s)**	**Year of Publication**	**Study Design**	**Study Location**	**Sample Size**	**Polymer Type**
**16**	Willi A, Patcas R, Zervou SK, Panayi N, Schätzle M, Eliades G, et al	2023	Experimental in vitro laboratory study.	Clinic of Orthodontics and Pediatric Dentistry, University of Zurich, Switzerland	5 samples analyzed via ATR-FTIR spectroscopy, 10 aligners immersed in distilled water for 1 week at 37°C.<br>Controls: 3 distilled water blanks (no aligners).	Tera Harz TC85A (Graphy, Seoul, Korea). Aliphatic urethane dimethacrylate (UDMA)-based photopolymer resin with acrylate/methacrylate functionalization.
	**Health Hazards/ health risks identified**	**Environmental Hazards/ impact**	**Data Quality**	**Methodological Approach**	**Results & Findings**	**Conclusion/ Recommendations**
	Urethane Dimethacrylate (UDMA) was leached from aligners into water (29–96 µg/L). No Bisphenol-A (BPA) detected (<0.25 µg/L).	Not directly assessed in this study. Leached monomers (e.g., UDMA) could contribute to environmental contamination if aligners or wash water are improperly disposed, but this was not quantitatively measured.	High-precision analytical methods (ATR-FTIR, LC–ESI-MS/MS, LC–APCI-MS/MS).<br>Triplicate analyses and calibration curves ensured repeatability and accuracy ($R^2 = 0.9995–0.9997$).<br>Small sample size and lack of long-term simulation.	ATR-FTIR Spectroscopy: To identify molecular structure and calculate degree of conversion (C=C bond reduction).<br>LC–MS/MS Analysis: To quantify leached monomers :<br>UDMA via LC–ESI-MS/MS (positive mode).<br>BPA via LC–APCI-MS/MS (negative mode.<br>Statistical Analysis: Descriptive statistics for DC% and compound concentrations.	The study reported a degree of conversion (DC%) of 83 ± 3.6%, indicating that the 3D-printed aligner resin underwent efficient polymerization with a high proportion of monomers converted to polymer. Bisphenol-A (BPA) was not detected in any sample (<0.25 µg/L), confirming the absence of this endocrine-disrupting compound in the material. However, urethane dimethacrylate (UDMA) was found to leach into water in concentrations ranging from 29.1 to 96.3 µg/L, with a mean value of 50.9 ± 15.6 µg/L. The results also showed high variability (~300%) between samples, which the authors attributed to potential inconsistencies in printing and post-curing procedures. Although the detected UDMA levels were below acute toxicity limits, the study highlighted a potential cytotoxic concern, as continuous and repeated exposure to such leachates considering aligners are worn daily and replaced frequently could cumulatively approach biologically [illegible]	The 3D-printed aligner resin is **efficiently polymerized and BPA-free** but shows **variable UDMA leaching** into water.<br>Repeated intraoral exposure to residual UDMA over multiple aligners may pose potential health hazards, warranting further investigation. Need for standardization of 3D printing processes and long-term biocompatibility testing under simulated oral conditions.
